# The enhanced genomic 6 mA metabolism contributes to the proliferation and migration of TSCC cells

**DOI:** 10.1038/s41368-022-00161-9

**Published:** 2022-02-17

**Authors:** Lei Xi, Ying Yang, Ying Xu, Fangming Zhang, Jinghui Li, Xiyang Liu, Zhenxi Zhang, Quan Du

**Affiliations:** 1 State Key Laboratory of Natural and Biomimetic Drugs, School of Pharmaceutical Sciences, Peking University, Beijing, China; 2 Department of Stomatology, Beijing Friendship Hospital, Capital Medical University, Beijing, China

**Keywords:** Oral cancer, Oral cancer, Cancer epigenetics

## Abstract

In contrast to the well-established genomic 5-methylcytosine (5mC), the existence of N^6^-methyladenine (6 mA) in eukaryotic genomes was discovered only recently. Initial studies found that it was actively regulated in cancer cells, suggesting its involvement in the process of carcinogenesis. However, the contribution of 6 mA in tongue squamous cell carcinoma (TSCC) still remains uncharacterized. In this study, a pan-cancer type analysis was first performed, which revealed enhanced 6 mA metabolism in diverse cancer types. The study was then focused on the regulation of 6 mA metabolism, as well as its effects on TSCC cells. To these aspects, genome 6 mA level was found greatly increased in TSCC tissues and cultured cells. By knocking down 6 mA methylases *N6AMT1* and *METTL4*, the level of genomic 6 mA was decreased in TSCC cells. This led to suppressed colony formation and cell migration. By contrast, knockdown of 6 mA demethylase *ALKBH1* resulted in an increased 6 mA level, enhanced colony formation, and cell migration. Further study suggested that regulation of the NF-κB pathway might contribute to the enhanced migration of TSCC cells. Therefore, in the case of TSCC, we have shown that genomic 6 mA modification is involved in the proliferation and migration of cancer cells.

## Introduction

In eukaryotic genomes, there are two forms of DNA methylation that have been characterized so far, namely 5-methylcytosine (5mC) and N^6^-methyladenine (6 mA). In contrast to the well-established 5mC methylation, exist of 6 mA methylation was only recently revealed in eukaryotic genomes including *Drosophila*, *Caenorhabditis elegans*, *Chlamydomonas*, plants, and mammals.^1–8^ As an aspect of the 6 mA study, its metabolic processes including methylation and demethylation were investigated. This led to the characterization of two 6 mA methylases (N6AMT1, METTL4) and one demethylase (ALKBH1).^5,8,9^ METTL4 was a recently identified 6 mA metabolic enzyme, which was independently characterized by Kweon^9^ and our group.^10^

Another important aspect of the 6 mA study is to explore its function in mammalian cells. To this end, we have examined its abundance and regulation in lung and liver cancer cells.^11^ The findings we made are as follows. (1) With a reference to genomic 5mC, the abundance of 6 mA was found only ~10% that of 5mC. (2) In contrast to the relative stable 5mC levels, dramatically decreased 6 mA levels were found with the cancer tissues relative to normal controls. (3) When 6 mA levels of the in vitro cultured cancer cells were examined, a great increase of 646-fold was revealed relative to the cancer tissues; while the increase was only 3.72-fold for 5mC levels. Together, our study characterized relatively low but dramatically regulated 6 mA levels in lung and liver cancer cells. Besides our study, changes of 6 mA level were also reported by other groups in different cancer types. The level of genomic 6 mA was found to decrease in gastric and liver cancer and to increase in glioblastoma and esophageal squamous cell carcinoma.^8,12,13^ Despite these contradictory changes, a common phenomenon was that genomic 6 mA was actively regulated in different cancer cells. These suggest the active roles of this novel epigenetic modification in the process of carcinogenesis.

To further examine this speculation, the present study was performed with tissues and cultured cells of tongue squamous cell carcinoma (TSCC). TSCC is the most common oral cancer type, which is characterized by its strong lymph node and distant metastasis capability.^14–16^ The incidence of the disease is rising in the population, particularly for women and/or young adults.^17–19^ Despite the recent advances in the therapeutic management of TSCC by combinatory strategy involving surgery, radiation, and chemotherapy, the mortality of TSCC is still at a high level.^20,21^ Thus, it is necessary to understand the molecular mechanism of TSCC progression and find novel therapeutic strategies for TSCC. Previous studies have shown that aberrant DNA methylation, including global DNA hypomethylation and gene promoter-associated CpG island hypermethylation, is observed in TSCC tissues and plays an important role in the tumorigenesis, development, and progression of TSCC.^22–24^ However, the role of 6 mA in TSCC has not been investigated yet.

In this study, we explored the function and mechanism of DNA 6 mA in TSCC. Our findings demonstrated that genomic 6 mA level was significantly increased in TSCC and DNA 6 mA promoted tongue cancer cells proliferation and migration. In addition, we identified migration-related genes regulated by 6 mA. These results suggested that 6 mA exerted a crucial role in TSCC by activating transcription of NF-κB pathway factors.

## Results

### The enhanced 6 mA metabolism in diverse cancer types

As an essential epigenetic process, genomic DNA metabolism consists of DNA methylation and demethylation. In the case of genomic 6 mA, two 6 mA methylases (N6AMT1, METTL4) and one 6 mA demethylase (ALKBH1) have been characterized. To resolve their evolution from prokaryotes to eukaryotes, phylogenetic tree analysis was performed using MEGA.^25^ In which, high evolutionary conservation was disclosed for N6AMT1, METTL4, and ALKBH1. On the one hand, this indicates that genomic 6 mA is an essential epigenetic modification; on the other hand, it proposes the active roles of these enzymes in diverse forms of life.

**Fig. 1:**
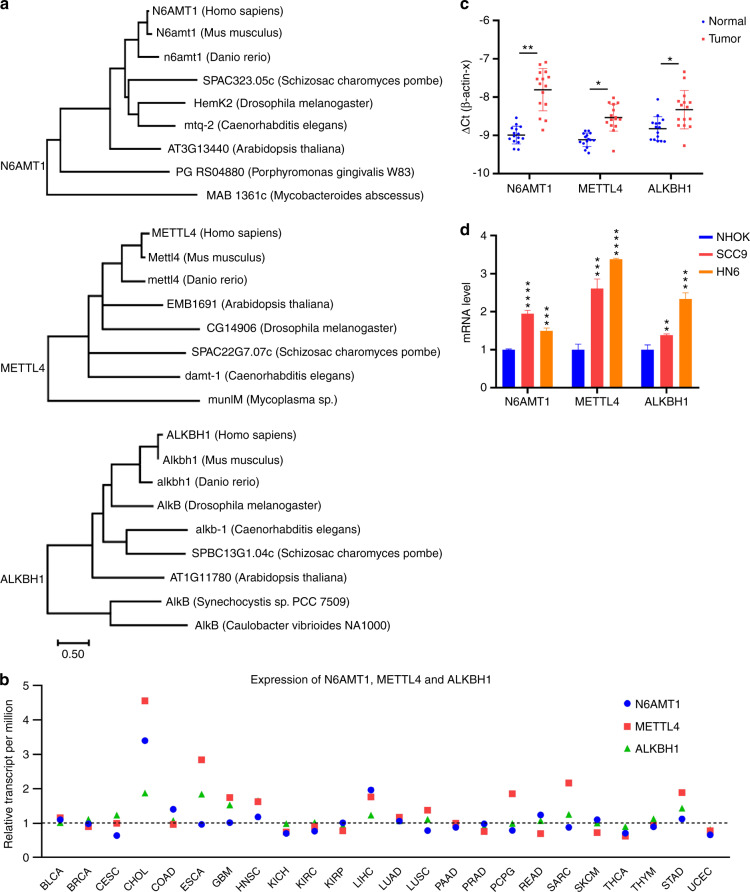
Up-regulated expression of 6 mA modification enzymes in TSCC tissues and cells. **a** Phylogenetic trees of 6 mA methylases *N6AMT1*, *METTL4*, and 6 mA demethylase *ALKBH1*. **b** Analysis of TCGA cancer data. In contrast to the respective normal control, the relative expression of *N6AMT1*, *METTL4*, and *ALKBH1* was individually calculated with all the cancer cases collected in the TGCG library. The data were then summarized in terms of cancer type. BLCA, 408 cases; BRCA, 1097; CESC, 305; CHOL, 36; COAD, 286; ESCA, 184; GBM, 156; HNSC, 520; KICH, 67; KIRC, 533; KIRP, 290; LIHC, 371; LUAD, 515; LUSC, 503; PAAD, 178; PRAD, 497; PCPG, 179; READ, 166; SARC, 260; SKCM, 472; THCA, 505; THYM, 120; STAD, 415; UCEC, 546. **c** The levels of *N6AMT1*, *METTL4*, and *ALKBH1* in tongue tumors and the adjacent normal tissues were collected from 15 patients. **d** The levels of *N6AMT1*, *METTL4*, and *ALKBH1* in NHOK, SCC9, and HN6 cells. The data are presented as mean ± SD (Student’s *t*-test, *n* = 3); *P < 0.05; **P < 0.01; ***P < 0.001

To explore their regulation in cancers, we carried out a pan-cancer type analysis, using cancer data collected in the TCGA database.^26^ Levels of the three enzymes were examined across 24 cancer types relative to their normal controls. This analysis revealed a general up-regulation of 6 mA metabolic enzymes in cancer tissues, although a specific change of an enzyme or the degree of the change were cancer-type- or cancer-tissue-dependent (Fig. 1b, Supplementary Fig. 1).

Then the expression of the enzymes was further examined with TSCC tissues collected in our study. RT-qPCR assay found that all the expression of *N6AMT1*, *METTL4*, and *ALKBH1* increased in TSCC tissues relative to the paired adjacent normal tissues (Fig. 1c). With cultured TSCC cells, similar regulation profiles were identified with SCC9 and HN6 cells. Primary human oral keratinocytes (NHOK) cells were included as normal control (Fig. 1d).

Therefore, consistent with the earlier studies,^8,12,13^ enhanced 6 mA metabolism was corroborated in diverse cancer types, as well as the TSCC tissues and cultured cells.

### Increased 6 mA levels in TSCC tissues and cells

**Fig. 2:**
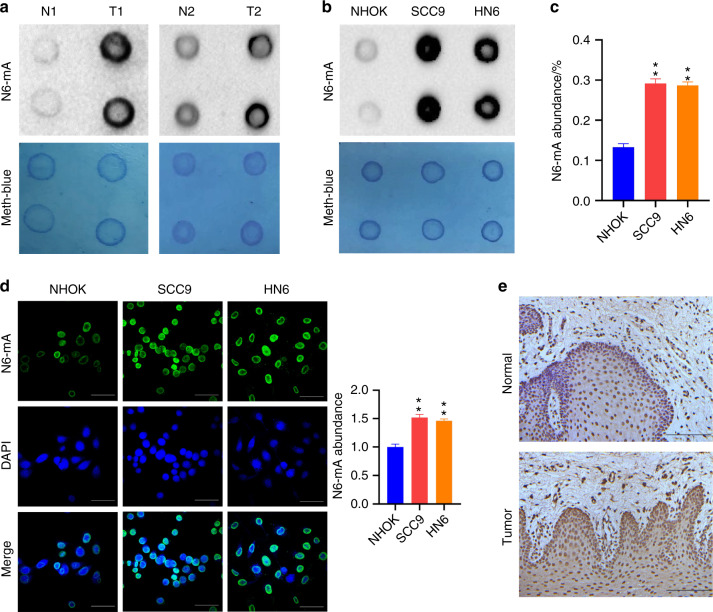
Elevated 6 mA levels in TSCC tissues and cells. **a** Genomic 6 mA levels of tongue tumor tissues (T) and control tissues (N) were examined by dot blot assay. **b**, **c** Genomic 6 mA levels of cultured cells were examined by dot blot assay (**b**) and HPLC-MS/MS assay (**c**). Sample loading was checked by Methylene blue staining. **d** With cultured cells, genomic 6 mA was detected by 6 mA immunofluorescent and quantified. Scale bar, 50 μm. **e** Immunohistochemistry staining of 6 mA was performed with tongue tumor tissues. Scale bar, 100 μm

To evaluate the effects of the enhanced 6 mA metabolism, a dot blot assay was performed with genomic DNA extracted from surgically isolated TSCC tissues. With a reference to the normal controls, increased 6 mA levels were identified (Fig. 2a). To confirm this observation, a dot blot assay was performed with SCC9 and HN6 cells, showing increased genomic 6 mA levels in cultured TSCC cells (Fig. 2b).

To quantitatively examine the regulation, 6 mA level was examined by means of HPLC-MS/MS assay. With SCC9, HN6, and NHOK cells, genomic DNAs were extracted. The DNAs were then subjected to the treatment of RNase A, DNase I, nuclease P1, and calf intestinal alkaline phosphatase, to degrade the polynucleotides to oligonucleotides, nucleotides, and nucleosides finally. The resulted nucleoside products were analyzed by HPLC-MS/MS. Using native dA and 6 mA as standards, the levels of dA and 6 mA were determined. The abundance of 6 mA was presented as a mole ratio relative to that of dA. Results showed that relative to NHOK, genomic 6 mA levels increased to about 2.2-fold for SCC9 or HN6 cells (Fig. 2c).

By means of immunofluorescent and immunohistochemical staining, the levels of 6 mA were further examined with cultured cells and tissue sections. Results showed that SCC9 and HN6 cells exhibited much higher 6 mA staining relative to that of NHOK cells (Fig. 2d), while increased 6 mA levels were detected with the tongue tumor tissues relative to the matched adjacent normal tissues (Fig. 2e). These results were consistent with the earlier data obtained with dot blot and HPLC-MS/MS analyses.

Therefore, in the case of TSCC, enhanced 6 mA metabolism up-regulates the levels of genomic 6 mA methylation.

### 6 mA regulation by N6AMT1, METTL4, or ALKBH1

**Fig. 3:**
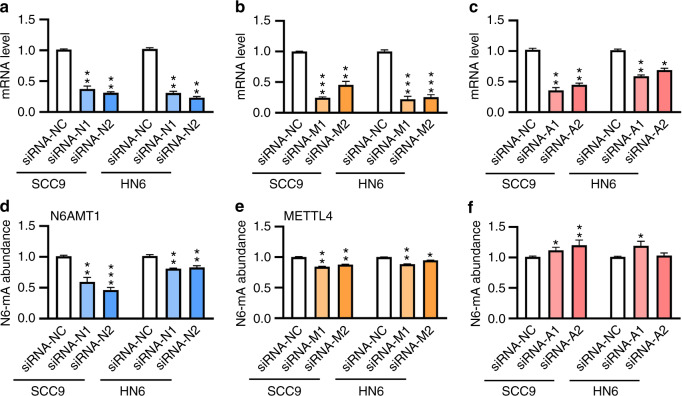
Characterization of 6 mA modification enzymes in cultured TSCC cells. Loss-of-function assays were performed with *N6AMT1* (**a**), *METTL4* (**b**), and *ALKBH1* (**c**) by siRNAs transfection. Knockdown of *N6AMT1* (**d**), *METTL4* (**e**), and *ALKBH1* (**f**) led to markedly regulated 6 mA levels in SCC9 and HN6 cells examined by HPLC-MS/MS

To explore the significance of 6 mA regulation, a loss-of-function assay was performed with these enzymes. To this aspect, gene-specific siRNAs were designed to target *N6AMT1*, *METTL4*, or *ALKBH1*. Transfection of these siRNAs into SCC9 or HN6 cells led to the downregulated expression of their respective target genes (Fig. 3a–c). As a consequence of *N6AMT1* knockdown by two independent siRNAs, 6 mA level was decreased to 45% and 60% in SCC9 cells, to 81% and 88% in HN6 cells (Fig. 3d, Supplementary Fig. 2a). Knockdown of *METTL4* decreased 6 mA level to 84% and 88% in SCC9 cells, to 89% and 95% in HN6 cells (Fig. 3e, Supplementary Fig. 2b). Knockdown of *ALKBH1* increased 6 mA level to 111% and 120% in SCC9 cells, to 103% and 120% in HN6 cells(Fig. 3f, Supplementary Fig. 2c).

For the two 6 mA methylases, knockdown of *N6AMT1* led to a greater decrease of 6 mA level in SCC9 cells relative to that of HN6 cells, while a comparable effect was found with *METTL4* knockdown cells. When *N6AMT1* and *METTL4* were simultaneously knocked down in the cells (Supplementary Fig. 3a, b), the synergic effect was however not observed in terms of the abundance of 6 mA modification (Supplementary Fig. 3c, d). Considering the differential regulation of the metabolic enzymes and 6 mA level, other issues were likely involved in the process, such as cell-specific factors, specific methylation sites of each enzyme.

### The effects on cell proliferation

Cancer cells are featured by many properties such as genome instability, infinite growth potential, insensitivity to antigrowth signal, resisting to cell death, as well as tissue invasion and metastasis.^27^ Among them, enhanced cell proliferation and migration activity are the most distinguishing feature of cancer cells.

**Fig. 4:**
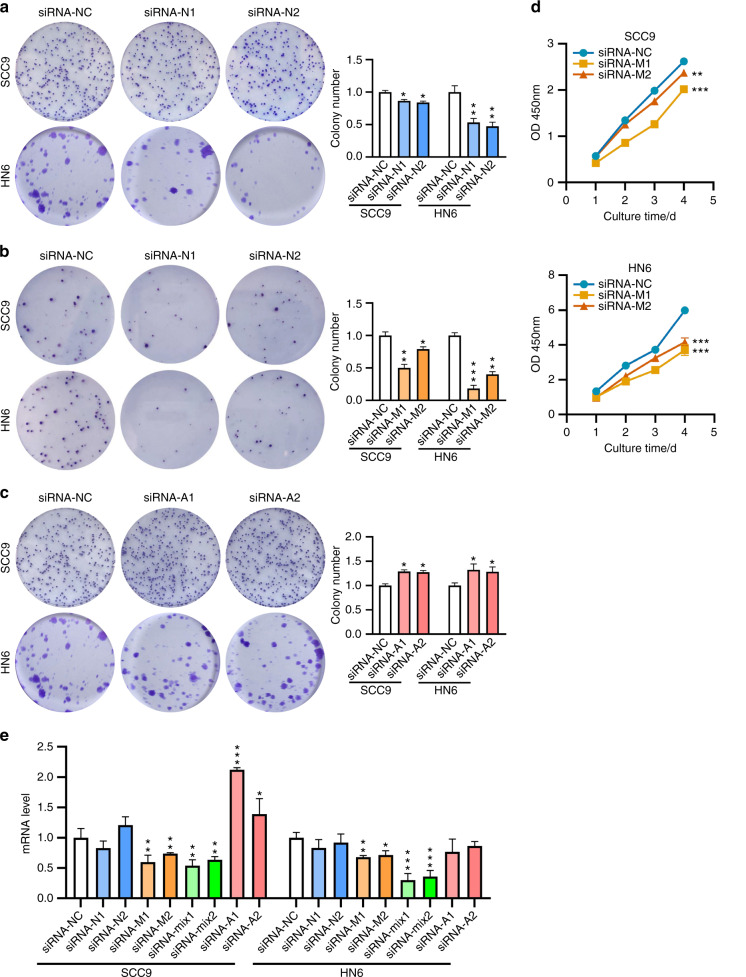
The effects of gene silencing on cell proliferation. Colony formation of *N6AMT1* (**a**), *METTL4* (**b**), and *ALKBH1* (**c**) knockdown cells were performed, both the images and quantified data are presented. **d** CCK-8 assay was performed with *METTL4* knockdown cells. **e** The effects on PCNA expression

To explore the influence on proliferation, colony formation, and CCK-8 assays were carried out with *N6AMT1*, *METTL4*, or *ALKBH1* knockdown cells. Results showed that for the *N6AMT1* or *METTL4* knockdown cells, decreased 6 mA level led to suppressed colony formation capability (Fig. 4a, b), while for *ALKBH1* knockdown cells, increased 6 mA level promoted colony formation (Fig. 4c).

CCK-8 assay found that decreased 6 mA level by *METTL4* knockdown led to significantly suppressed cell proliferation (Fig. 4d), while decreased 6 mA level by *N6AMT1* knockdown had only a marginal effect on cell proliferation (Supplementary Fig. 4a, b). Interestingly, when the 6 mA level was increased by *ALKBH1* knockdown, no evident effect was observed (Supplementary Fig. 4c, d). To corroborate these findings, expression of the proliferating cell nuclear antigen (PCNA, an indicator of cell viability) was examined. We found that down-regulation of 6 mA level by *METTL4* knockdown suppressed *PCNA* expression, while 6 mA downregulation by *N6AMT1* knockdown or up-regulation by *ALKBH1* knockdown had no effect on *PCNA* expression (Fig. 4e).

Taken together, these results indicate that genomic 6 mA modification is involved in the cell proliferation of TSCC cells. While the general 6 mA status correlates with colony formation capability, the proliferation is more likely affected by specific genes that are differentially regulated by these enzymes.

### The effects on cell migration

**Fig. 5:**
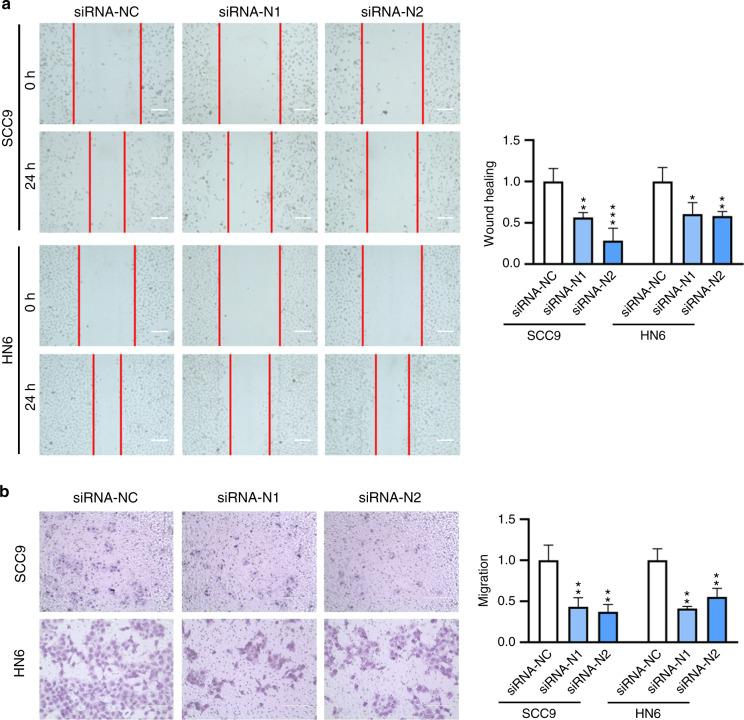
Knockdown of 6 mA methylase N6AMT1 led to inhibited cell migration. **a** Wound healing assay of *N6AMT1* knockdown cells. Cell images were taken and analyzed at 0 and 24 h after transfection. Scale bar, 200 μm. **b** Transwell assay of *N6AMT1* knockdown cells. Cell images were taken and analyzed at 24 h after transfection. Scale bar, 200 μm

**Fig. 6:**
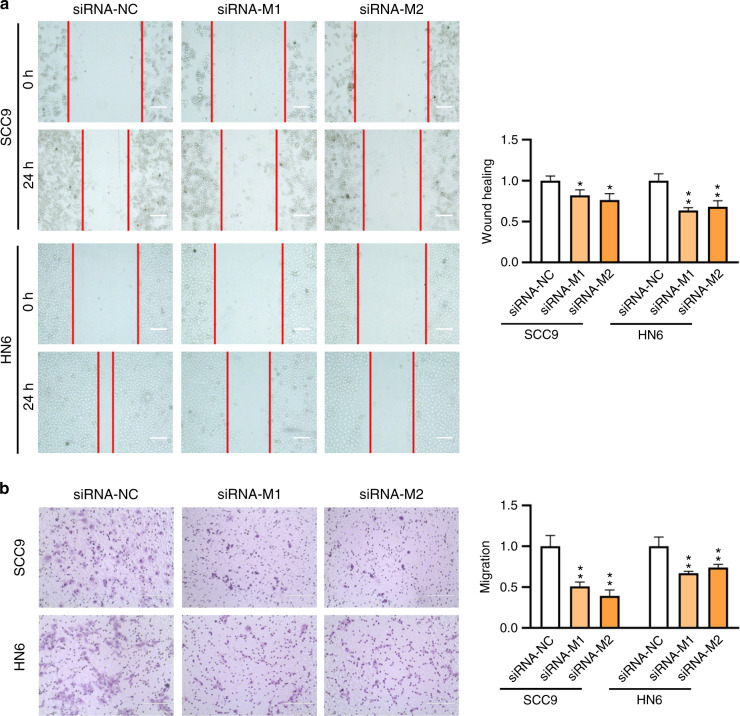
Knockdown of 6 mA methylase METTL4 led to inhibited cell migration. **a** Wound healing assay of *METTL4* knockdown cells. Cell images were taken and analyzed at 0 and 24 h after transfection. Scale bar, 200 μm. **b** Transwell assay of *METTL4* knockdown cells. Cell images were taken and analyzed at 24 h after transfection. Scale bar, 200 μm

**Fig. 7:**
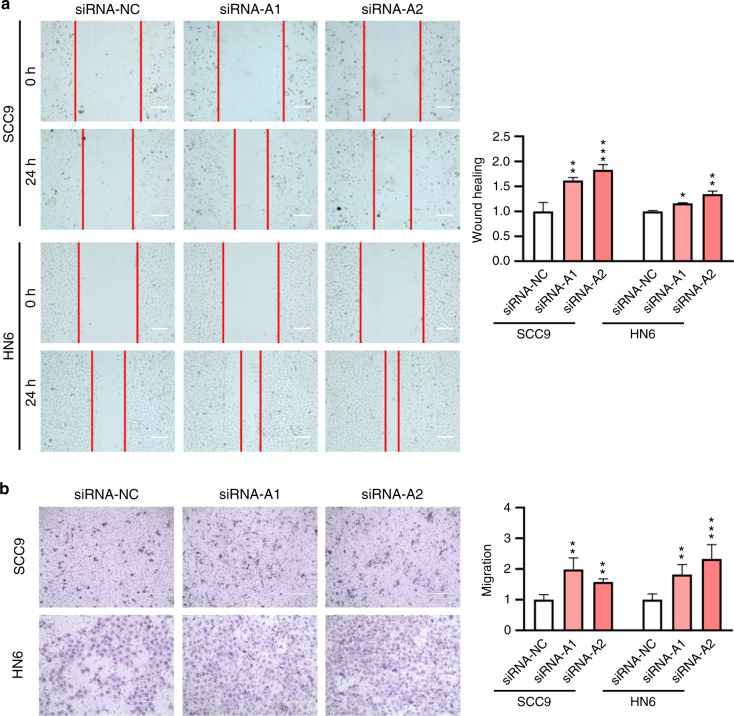
Knockdown of 6 mA demethylase ALKBH1 led to increased cell migration. **a** Wound healing assay of *ALKBH1* knockdown cells. Cell images were taken and analyzed at 0 and 24 h after transfection. Scale bar, 200 μm. **b** Transwell assay of *ALKBH1* knockdown cells. Cell images were taken and analyzed 24 h after transfection. Scale bar, 200 μm

To investigate the effect on cell migration, a wound-healing assay was performed with *N6AMT1*, *METTL4*, or *ALKBH1* knockdown cells. In brief, when the cells grew to sub-confluent, a wound gap was manually made by scratch. The healing of the wound was monitored by microscopic observation and measurement. It was shown that when *N6AMT1*, *METTL4* was knocked down individually or combinedly, prolonged wound closure was resulted (Fig. 5a, Fig. 6a, Supplementary Fig. 5a). By contrast, the knockdown of *ALKBH1* promoted the process of wound closure (Fig. 7a). Therefore, upregulating 6 mA modification promoted cell migration, and downregulating 6 mA inhibited cell migration.

In addition to wound healing, a transwell assay was also performed with the cells. Results showed that downregulation of 6 mA level led to repressed cell migration (Figs. 5b, Fig. 6b, Supplementary Fig. 5b), while upregulation of 6 mA level led to enhanced cell migration (Fig. 7b).

Similarly, a synergic effect was not observed with *N6AMT1* and *METTL4*.

### The NF-κB pathway is positively regulated by 6 mA methylation

To characterize the genes that contribute to the migration of TSCC cells, a candidate gene set was selected from the literature. They are five matrix metalloproteinases (*MMP1*, *MMP2*, *MMP9*, *MMP11*, *MMP13*),^28–32^ three EMT-relevant genes (*N-cadherin*, *Vimentin*, *β-catenin*)^33–35^ and two NF-κB pathway factors (*p65*, *uPA*).^36–39^ Expression of these genes was first examined with the head and neck squamous cell (HNSC) data of the TCGA database relative to their control. The result showed a generally increased profile in HNSC cancers (Supplementary Fig. 6a).

Their expression was then examined with TSCC tissues collected in the study, by means of RT-qPCR. To our surprise, all the MMPs were down-regulated substantially (Supplementary Fig. 6b). Consistently, their expression in SCC9 cells decrease to a level that was hardly detected. Because of their activity in the degradation of extracellular matrix and basement membranes, MMPs were generally thought to contribute to the aggressiveness of cancer cells. However, in recent studies, MMPs were also found to provide beneficial and protective effects in cancer progression.^40–43^ The diverse activity of MMPs may explain the observation in our study.

**Fig. 8:**
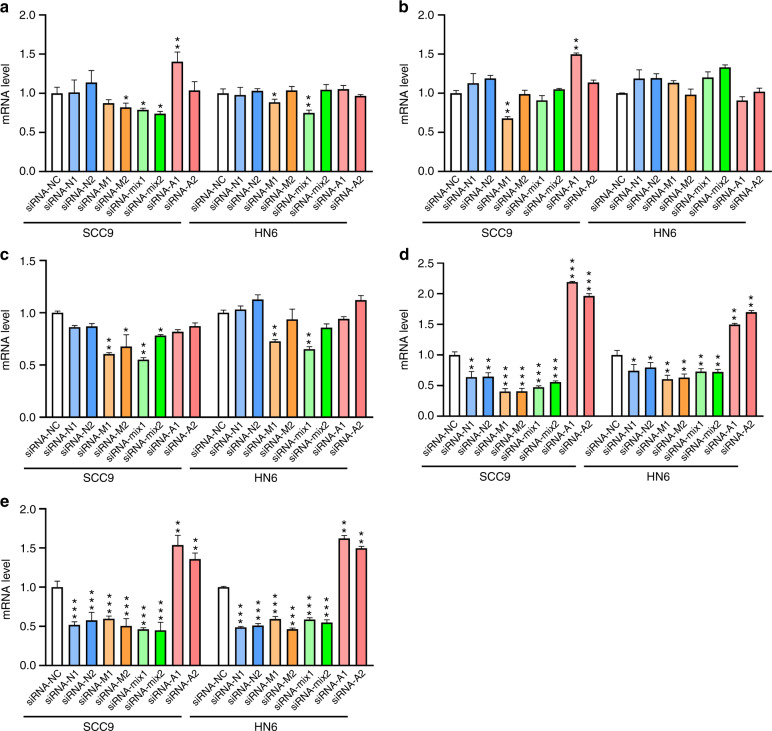
Screening of migration-related genes. siRNAs targeting 6 mA methylase *N6AMT1*, *METTL4, ALKBH1,* or both *N6AMT1* and *METTL4* were transfected into SCC9 or HN6 cells. The effects on gene expression of a candidate set *N-cadherin* (**a**), *Vimentin* (**b**), *β-catenin* (**c**), *p65* (**d**), *uPA* (**e**) were examined by RT-qPCR

In contrast to MMPs, increased expression of *N-cadherin*, *Vimentin*, *β-catenin*, *p65*, and *uPA* was revealed with TSCC tissues (Supplementary Fig. 6b). To further examine their contribution to cell migration, the expression of these genes was investigated with migration-compromised SCC9 or HN6 cells (Fig. 8a–e). We found that down-regulation of 6 mA led to stably decreased expression of *p65* and *uPA* for both cells, while up-regulation of 6 mA led to stably increased expression of these genes (Fig. 8d, e) together with compromised cell migration activity. Therefore, regulation of the NF-κB pathway is likely involved in the enhanced migration of TSCC cells.

### Rescue assay of *ALKBH1* knockdown cells

**Fig. 9:**
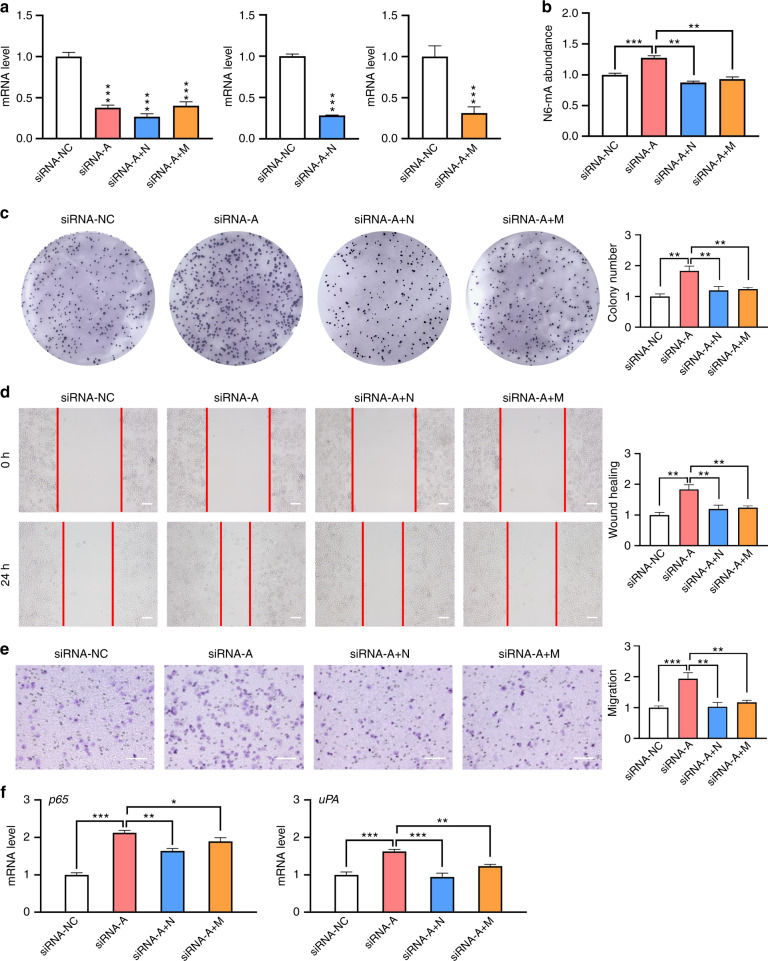
Rescue assays performed with SCC9 cells. **a** Gene knockdown of *ALKBH1*, or double knockdown of *ALKBH1* + *N6AMT1, ALKBH1* + *METTL4*. **b** 6 mA levels of single or double-knockdown cells were examined by HPLC-MS/MS. **c** Colony formation of gene knockdown cells. Both the images and quantified data are presented. **d** Wound healing of gene knockdown cells. Scale bar, 100 μm. **e** Transwell assay of gene knockdown cells. Scale bar, 100 μm. **f** Gene expression of *p65* and *uPA*

To further corroborate these findings, rescue assays were performed with ALKBH1 knockdown cells. Gene-specific siRNAs were transfected to knock down the expression of 6 mA modification enzymes *ALKBH1*, *ALKBH1* + *N6AMT1*, and *ALKBH1* + *METTL4* in SCC9 cells (Fig. 9a) and HN6 cells (Supplementary Fig. 7a), respectively. The increased level of genomic 6 mA induced by *ALKBH1* knockdown was rescued by double knocking down with *ALKBH1* + *N6AMT1* or *ALKBH1* + *METTL4* (Fig. 9b; Supplementary Fig. 7b). With simultaneous knockdown of 6 mA methylases *N6AMT1* or *METTL4*, phenotypes of *ALKBH1* knockdown cells were significantly rescued by colony formation assay (Fig. 9c; Supplementary Fig. 7c), wound healing assay (Fig. 9d; Supplementary Fig. 7d), and transwell assay (Fig. 9e; Supplementary Fig. 7e). The gene expression of two downstream factors, *p65* and *uPA*, were also changed consistently (Fig. 9f; Supplementary Fig. 7f). Therefore, these rescue experiments provided stronger evidence that genomic 6 mA modification was involved in the proliferation and migration of the TSCC cells.

## Discussion

It is widely recognized that aberrant genomic DNA modification is critically important to the development and progress of cancers.^44^ Recurrent 5mC hypomethylation of cancer genome provides not only biomarkers for cancer screening, but also therapeutic targets in clinical practice.^45^ Following the characterization of 6 mA modification in eukaryotic genomes, attention has been drawn to its implications in tumorigenesis. Recent studies found that genomic 6 mA modification was downregulated in gastric and liver cancer, and markedly upregulated in glioblastoma and esophageal squamous cell carcinoma.^8,12,13^ Although cancer-type-specific 6 mA level changes were revealed in these studies, metabolism of 6 mA was shown to be generally enhanced in diverse cancer types. In agreement with these studies, the pan-cancer analysis performed in the present study revealed a general upregulation of 6 mA metabolic enzymes in cancer tissues relative to their normal controls.

With mouse embryonic stem cells, Alkbh1 was characterized as a 6 mA demethylase as knockout of *ALKBH1* led to increased 6 mA level.^5^ The Alkbh1-deficient cells show transcriptional silencing of young LINE-1 transposon elements. Investigation of *ALKBH1* in glioblastoma indicated that *ALKBH1* functioned as a 6 mA demethylase, knockdown of *ALKBH1* led to inhibited tumor growth and compromised stemness and tumorigenesis, by means of downregulating the expression of hypoxia- and tumor-related genes.^12^ In which, no 6 mA methyltransferase activity has been detected either in vivo or in vitro. However, the study of Xiao et al.^8^ reported that *N6AMT1* was an essential 6 mA methyltransferase in liver and gastric cancers. To the aspect of METTL4, the study of Kweon et al.^9^ reported that Mettl4 catalyzed 6 mA deposition in genomic DNA, inactivation of METTL4 resulted in diminished 6 mA levels in mouse cells. Deposition of 6 mA by Mettl4 also triggers proteolytic destruction of sensor proteins (e.g., *ASXL1*) that have been linked to multiple cancers, including leukemia and glioblastoma. Hao et al. reported that METTL4 could mediate mammalian mtDNA 6 mA methylation, thus affecting mitochondrial transcription, replication, and activity.^46^

In this study, regulation and the activity of 6 mA metabolic enzymes were investigated with human TSCC tissues and cultured cells. In which, up-regulated expression of 6 mA metabolic enzymes and increased 6 mA levels were revealed in cancer tissues, suggesting their involvement in cancer development. To characterize the effects of the regulation, a loss-of-function assay was performed with these enzymes. Results showed that N6AMT1 and METTL4 deposited 6 mA modification on genomic DNA, while ALKBH1 removed 6 mA from it. However, no evident synergic effect was observed between the methylases, N6AMT1, and METTL4, suggesting other enzymes likely contribute to the process. Considering the DNA anchoring roles of genomic 6 mA modification, it is of interest to further characterize the 6 mA interacting factors that are responsible for triggering the downstream pathways.^47^ For the effects on colony formation and cell migration, we found that upregulated 6 mA levels promoted cell proliferation and migration, and down-regulation of 6 mA levels led to an opposite consequence.

Published studies indicated that the effects on gene activation or suppression depended on the position of 6 mA methylation.^48,49^ Therefore, the specific genes regulated by 6 mA might explain the cancer-type-relevant regulation. To this aspect, downstream pathway study was investigated in terms of cell proliferation and migration. The result showed that *p65* and *uPA*, the factors of the NF-κB pathway, were positively regulated by 6 mA modification. The other migration-related genes were not affected. To obtain a more comprehensive gene regulation network, RNA sequencing (RNA-seq) combined with single-molecule, real-time sequencing can be performed to interrogate the effects of 6 mA on the whole transcriptome.

In conclusion, we report that the genomic 6 mA levels are elevated in TSCC tissues and are dynamically regulated in TSCC cell lines by DNA methylases N6AMT1, METTL4, and demethylase ALKBH1 and that maintenance of this regulatory circuitry is critical for cell proliferation and migration. Our findings reveal a new class of DNA modification in TSCC and identify a potential target that can be exploited for cancer therapy.

## Materials and methods

### Clinical sample collection

With the approval of the ethical committee of Beijing Friendship Hospital, Capital Medical University (2018-P2-214-01), tongue tumors and the adjacent normal tissues were collected from 15 patients. All the patients received radical surgery without any form of presurgical adjuvant therapy. Informed consent was provided by the patients for the use of their tissues and data. Clinicopathologic features of the patients are summarized in Table 1.

**Table 1:** Clinicopathologic features of the TSCC samples in the present study

Sample number	Patient sex	Patient age (Years)	Pathological diagnosis	pTNM stage
1	Female	72	TSCC (moderately differentiated)	pT2N0M0
2	Female	67	TSCC (highly to moderately differentiated)	pT2N2bM0
3	Female	63	TSCC (well differentiated)	pT1N0M0
4	Male	79	TSCC (moderately differentiated)	pT2N0M0
5	Female	63	TSCC (highly to Moderately differentiated)	pT2N2bM0
6	Female	87	TSCC (moderately differentiated)	pT1N0M0
7	Female	63	TSCC (moderately differentiated)	pT2N0M0
8	Male	54	TSCC (moderately differentiated)	pT3N0M0
9	Male	54	TSCC (well differentiated)	pT2N0M0
10	Female	73	TSCC (well differentiated)	pT2N0M0
11	Female	49	TSCC (highly to moderately differentiated)	pT1N2bM0
12	Female	80	TSCC (well differentiated)	pT1N0M0
13	Male	72	TSCC (well differentiated)	pT2N0M0
14	Female	77	TSCC (well differentiated)	pT1N0M0
15	Male	33	TSCC (moderately differentiated)	pT2N2bM0

### Cell culture

SCC9 cells were maintained in Dulbecco’s Modified Eagle Medium: F-12 (Invitrogen), supplemented with 10% FBS (Invitrogen) and 400 ng·mL^−1^ hydrocortisone. HN6 cells were maintained in Dulbecco’s Modified Eagle’s Medium (Invitrogen), supplemented with 10% fetal bovine serum (FBS). Primary NHOK were isolated from normal gingival tissues and cultured in the same medium as that of SCC9 cells. All the cell lines were cultured in a humidified atmosphere of 5% CO_2_ at 37 °C, and the medium was changed every 2 days.

### Gene silencing assay

Gene-specific siRNAs were chemically synthesized by RiboBio (Guangzhou, China). siRNAs were transfected into cultured cells using Lipofectamine^™^ RNAiMax Regent (Invitrogen), according to the manufacturer’s instruction. Two siRNAs targeting *N6AMT1* are as follows: siRNA-N1, 5′-GAACUGGCAGGAGUGGAAAdTdT; siRNA-N2, 5′-CCUCAAGUUCACCAAGUCUdTdT. Two siRNAs targeting *METTL4* are as follows: siRNA-M1, 5′-GGAAGAGGAUACAUCUGUUdTdT, 5′-GCAUUUGCCUUCUCUGAAUdT dT. Two siRNA-mixes targeting both *N6AMT1* and *METTL4* are as follows: siRNA-mix1: siRNA-N1 + siRNA-M1; siRNA-mix2: siRNA-N2 + siRNA-M2. Two siRNAs targeting *ALKBH1* are as follows: 5′-GCAAGCCUAUGGACUCAAAdTdT, 5′-GGAAUCCACGUAGACAGAUdTdT.

siRNA-mixes for *ALKBH1* + *N6AMT1* double-knockdown are as follows: siRNA-A + N: siRNA-A1 + siRNA-N2. siRNA-mixes for *ALKBH1* + *METTL4* double-knockdown are as follows: siRNA-A + M: siRNA-A1 + siRNA-M1.

A negative control siRNA was included in the study: siRNA-NC (siN0000001-1-5).

### Real-time quantitative PCR (RT-qPCR)

Total RNA was isolated using the TRIzol reagent (Invitrogen). Complementary DNA was synthesized using Hiscript II Q RT SuperMix for the qPCR kit (Vazyme). Quantitative PCR was performed using SYBR Green GoTaq qPCR Master Mix (Promega) and an MX3005P Real-Time PCR System (Agilent Technologies). The level of gene expression was quantified using the 2^−ΔΔCt^ method and normalized to that of β-actin. Sequences of the primers are listed in Supplementary Table 1.

### Dot blot assay

Genomic DNA was extracted using TIANamp Genomic DNA Kit (TIANGEN), treated by RNase A overnight at 50 °C, and purified using Universal DNA Purification Kit (TIANGEN). Purified DNA samples were denatured at 95 °C for 10 min, cooled down on the ice for 3 min, and then loaded at an amount of 200 ng per dot on Amersham Hybond-N^+^ membrane (GE). Membranes were air-dried for 5 min, and UV-crosslinked for 2 min. Membranes were blocked in TBST buffer containing 5% nonfat milk at room temperature for 1 h, incubated with anti-6mA antibody solution (1:2000, Synaptic Systems) at 4 °C overnight, and then incubated with HRP-conjugated anti-rabbit IgG antibody (1:3000, Abcam) at room temperature for 2 h. Signal detection was performed with an Immobilon Western Chemiluminescent HRP Substrate (Merck Millipore).

### Enzymatic hydrolysis of genomic DNA

Two micrograms purified DNA samples were treated by 8U DNase I at 37 °C for 12 h, 8 U Nuclease P1 at 50 °C for 12 h, and 1 U calf intestinal alkaline phosphatase (CIP) at 37 °C for 12 h. The hydrolysis products were dried in a vacuum centrifugal concentrator and washed with acetonitrile. The resulting nucleoside-containing fractions were redissolved in ultrapure water to a final concentration of 2 mg·mL^−1^ before HPLC-MS/MS assay.

### HPLC-MS/MS assay

10 mL Nucleosides sample was added to a 10 mL 6-Cl-Purine solution (3 mg·mL^−1^) and filtered through a 0.22 mm filter. 5 mL of the solution was injected into an LC-Ion Trap. DNA methylation was analyzed with an LC-ESI-MS/MS system consisting of a Shimadzu LC-20A HPLC system (Shimadzu, Kyoto, Japan) and an ABSciex QTRAP 5500 (AB Sciex, Canada). Data acquisition and processing were performed using AB SCIEX Analyst 1.5.2 Software (Applied Biosystems, CA). LC separation was performed using an Atlantis T3 column (4.6 mm × 150 mm, 5 mm, Waters) (Waters Corp., MA, USA) with a flow rate of 0.3 mL per min at 40 °C. 3 mmol·L^−1^ ammonium formate (solvent A) and methanol (solvent B) were used as mobile phases. A gradient of 2 min 2% B, 4 min 2%–5% B,2 min 5%–10% B, 4 min 10% B, 0.1 min 10%–95% B, 2 min 95% B, 0.1 min 95%–2% B, 4.9 min 2% B was used. Positive electrospray ionization mode was used to perform the mass spectrometry detection. Target analytes were monitored by multiple reaction monitoring (MRM) mode using the mass transitions (precursor ions/productions) of dC (228.1/112.1), dT (243.2/127.1), dA (252.2/136.0), dG (268.1/152.1), 5mC (242.0/126.0), 6 mA (266.1/150.1), and 6-Chloropurine (287.0/155.0). The MRM parameters of all nucleosides were optimized to achieve maximal detection sensitivity. Quantification was performed by comparing with standard curves. A relative ratio was calculated for each nucleoside, based on the calculated molar concentrations.

### Immunofluorescent staining

Cells were fixed in 4% paraformaldehyde and permeabilized in PBS containing 0.3% Triton X-100 for 15 min. After permeabilization, samples were treated with 2 N HCl for 30 min and subsequently neutralized for 10 min with 0.1 M sodium borate buffer pH 8.5. Then, the samples were blocked for 2 h in blocking solution (5% donkey serum in PBS, RNase A was added to a final concentration of 100 mg·mL^−1^), followed by incubation with 6 mA antibodies (1:200, Synaptic Systems). Primary antibodies were incubated overnight at 4 °C, followed by anti-rabbit Alex 488 (1:200; Invitrogen Molecular Probes, Eugene, OR) conjugated secondary antibodies for 1 h at room temperature. Nuclei were stained with DAPI. Images were taken using a confocal microscope (FV 300, Olympus).

### Immunohistochemistry (IHC)

DNA 6 mA levels were investigated by IHC in TSCC tissues. Briefly, formalin-fixed, paraffin-embedded tongue tumors and the adjacent normal tissues were immunostained using 6 mA antibodies (1:100, Synaptic Systems) for 20 h at 4 °C. After rinsing, the sections were incubated with the biotinylated secondary antibody (SPN9001, ZSGB-Bio) for 60 min at room temperature. Staining was visualized using 3, 30-diaminobenzidine (DAB) chromogen (Dako, Carpinteria, CA), and counterstaining was using hematoxylin before dehydration for 30 s.

### Colony formation assay

Twenty-four-hour after siRNA transfection, 1000 cells per dish were plated and cultured in 6 cm dishes for 2 weeks with medium replacement every 3 days. At the end of the culture, the cells were stained with 0.4% crystal violet solution for colon formation counting under a microscope. A clone is defined as a group of cells over 50.

### CCK-8 assay

Twenty-four-hour after siRNA transfection, 5 × 10^3^ cells per well were plated and cultured in 96-well plates. At indicated time points, 10 μL of cell-counting kit-8 (CCK-8) solution was added to each well and incubated for another 3 h. At the end of incubation, the cells were analyzed with an ELISA reader (Infinite 200 PRO multimode reader, Switzerland) at 450 nm, to calculate the profiles of cell proliferation.

### Wound-healing assay

Twenty-four-hour after siRNA transfection, 3 × 10^5^ cells per well were plated in 6-well plates and cultured to confluence. The cell monolayer was gently scratched using a 200 μL plastic pipette tip. Photomicrographs were taken by EVOS at 0 and 24 h time points to monitor the wound-healing process. The wound coverage percentage = (0 h width − 48 h width)/(0 h width) × 100%.

### Transwell migration assay

Transwell migration assay was performed using 24-well Transwell chambers (8 µm, Corning Inc). In brief, 24 h after siRNA transfection, 1 × 10^5^ cells were seeded into the upper chamber with 200 µL serum-free DMEM, and 500 µL DMEM containing 10% FBS was added to the lower chamber. Twenty-four-hour later, cells maintained within the upper chamber were wiped up. The filters were removed, fixed with 4% paraformaldehyde, and stained with 0.1% crystal violet. The numbers of migration cells were counted from five randomly chosen fields.

### Statistical analysis

Analyses were performed with GraphPad Prism8 software. Data are presented as mean ± SD. ANOVA and paired Student’s *t*-test were used to evaluate the statistical significance.

## Supplementary information

Supplementary Figures and supplementary table 1

read me
