# Supplementary material for: The enhanced genomic 6 mA metabolism contributes to the proliferation and migration of TSCC cells
Source: Int J Oral Sci. 2022 Feb 17;14:11. doi: 10.1038/s41368-022-00161-9 (PMC8854414; doi:10.1038/s41368-022-00161-9)
Supplement: Supplementary file 1 — Supplementary Figures and supplementary table 1 [file 41368_2022_161_MOESM1_ESM.docx]

**
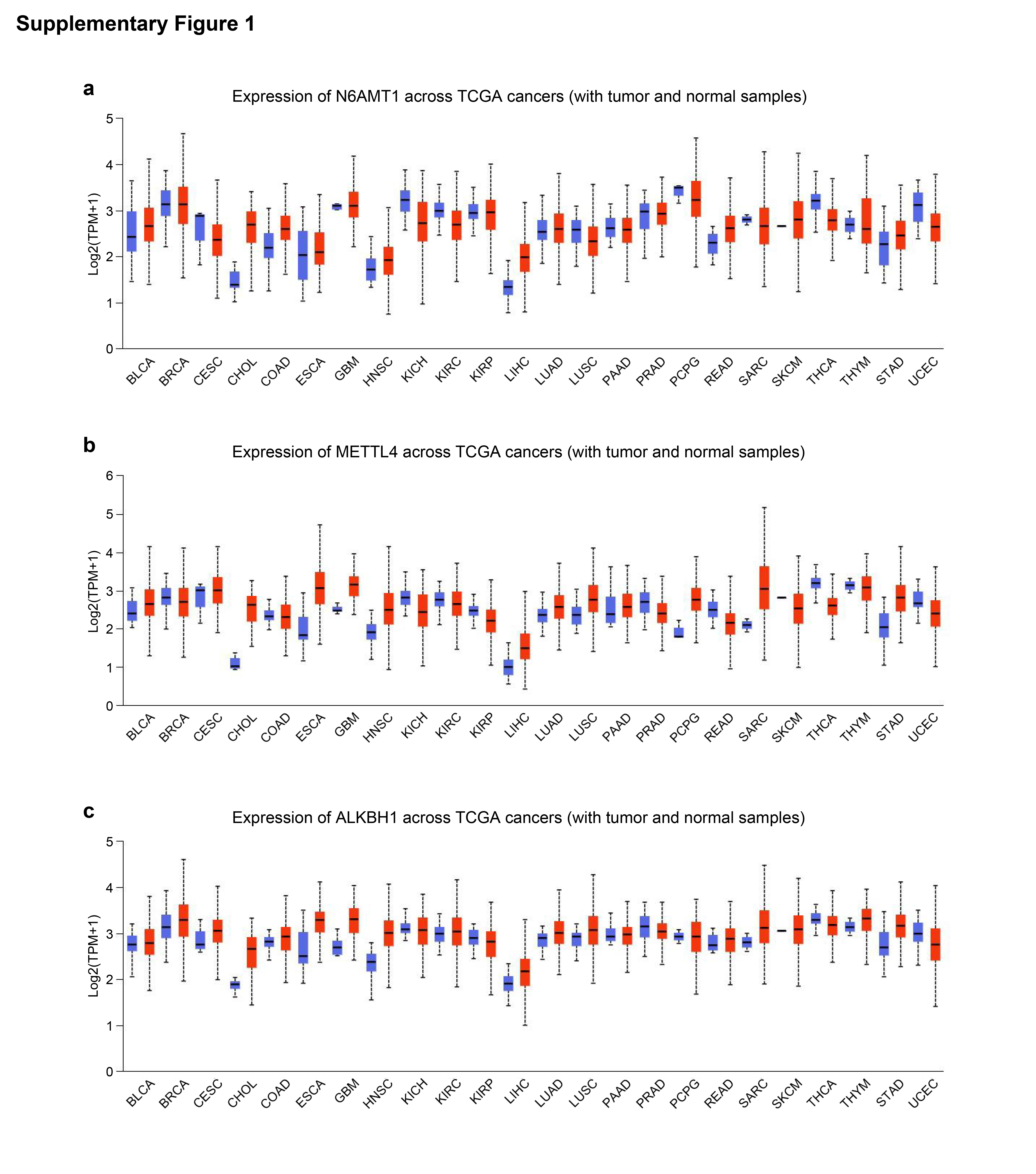
**

**Supplementary Figure 1.** Expression levels of *N6AMT1* (a), *METTL4* (b) and *ALKBH1* (c), across a variety of TCGA cancer types. Blue, normal tissue; red, tumor tissue.


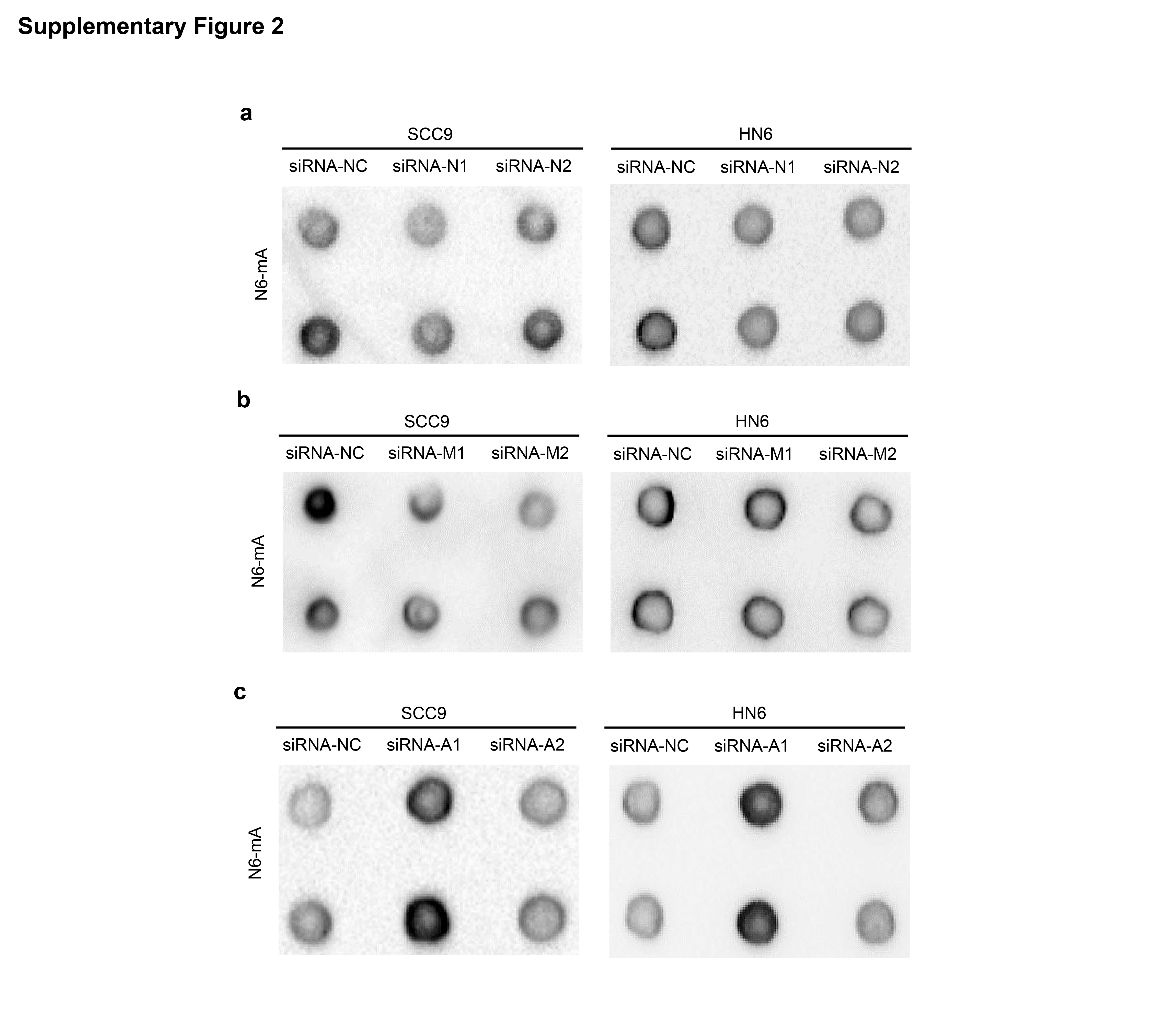


**Supplementary Figure 2.** Knockdown of 6mA modification enzymes led to modified genomic 6mA levels in cultured TSCC cells. (a) Knockdown of *N6AMT1* led to decreased genomic 6mA levels in SCC9 and HN6 cells which was examined by dot blot. (b) Knockdown of *METTL4* led to decreased 6mA levels. (c) Knockdown of *ALKBH1* led to increased 6mA levels.


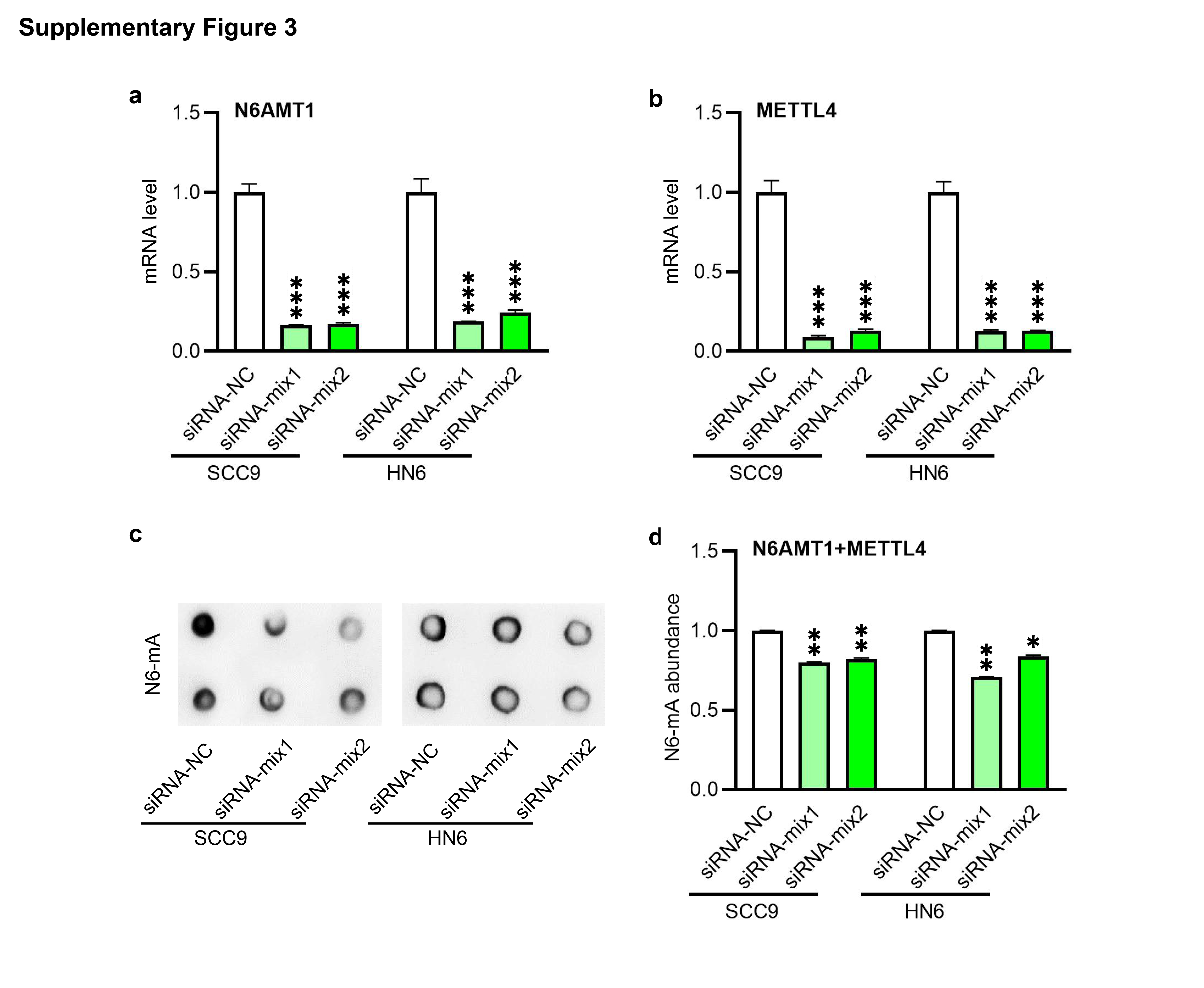


**Supplementary Figure 3.** Double knockdown of *N6AMT1* and *METTL4* decreased genomic 6mA levels of cultured cells. Gene knockdown of *N6AMT1* (a), or *METTL4* (b) was performed with SCC9 and HN6 cells. Genomic 6mA levels were examined by dot blot (c) and HPLC-MS/MS (d). siRNA-NC: sequence-irrelevant siRNA control; siRNA-mix1: siRNA-N1+siRNA-M1; siRNA-mix2: siRNA-N2+siRNA-M2. The data are presented as mean ± SD (student’s t-test, n = 3); *, p < 0.05; **, p < 0.01; ***, p < 0.001.

**
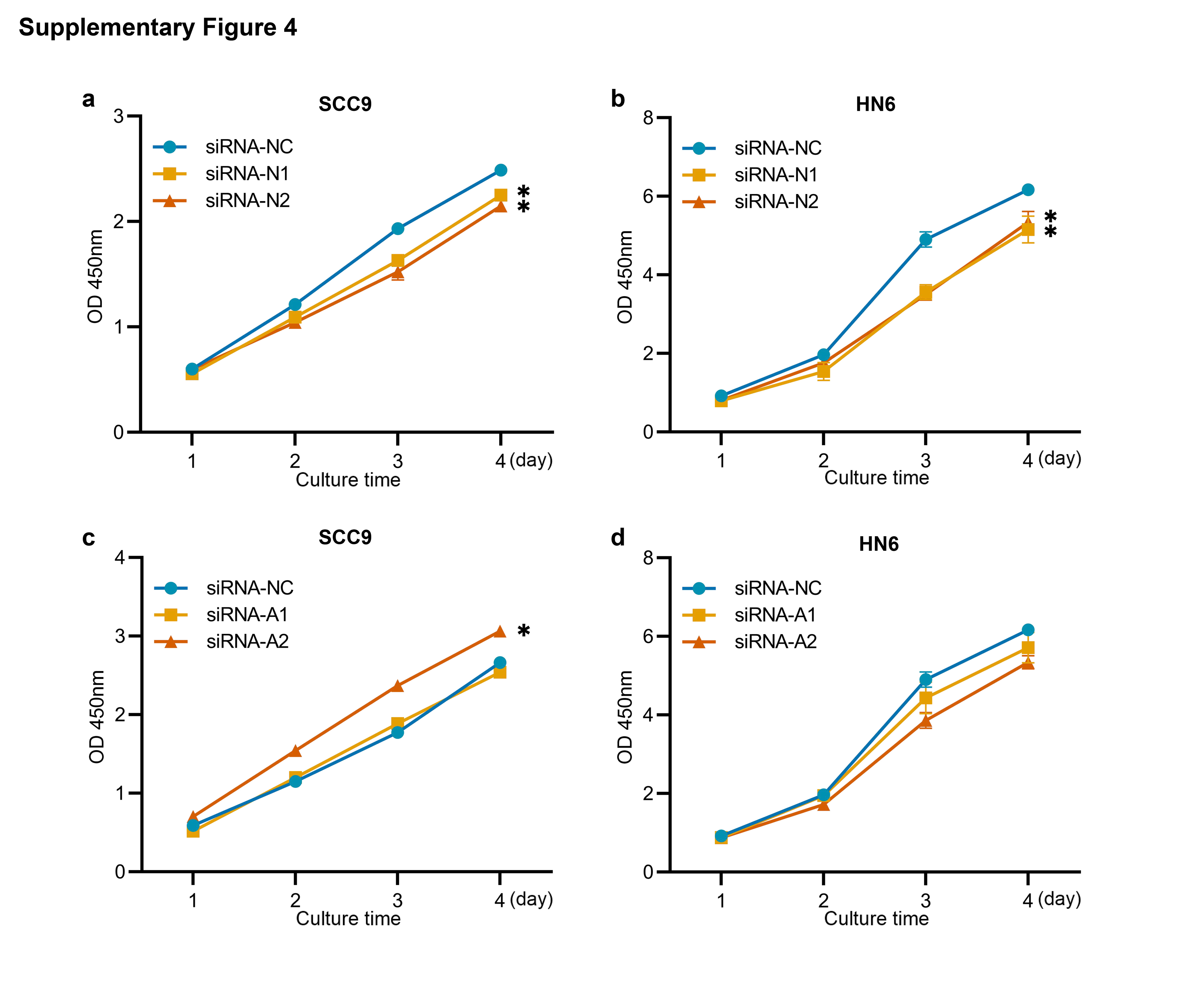
Supplementary Figure 4.** CCK-8 assays were performed with *N6AMT1* knockdown SCC9 (a) or HN6 cells (b), as well as *ALKBH1* knockdown SCC9 (c) or HN6 (d) cells.


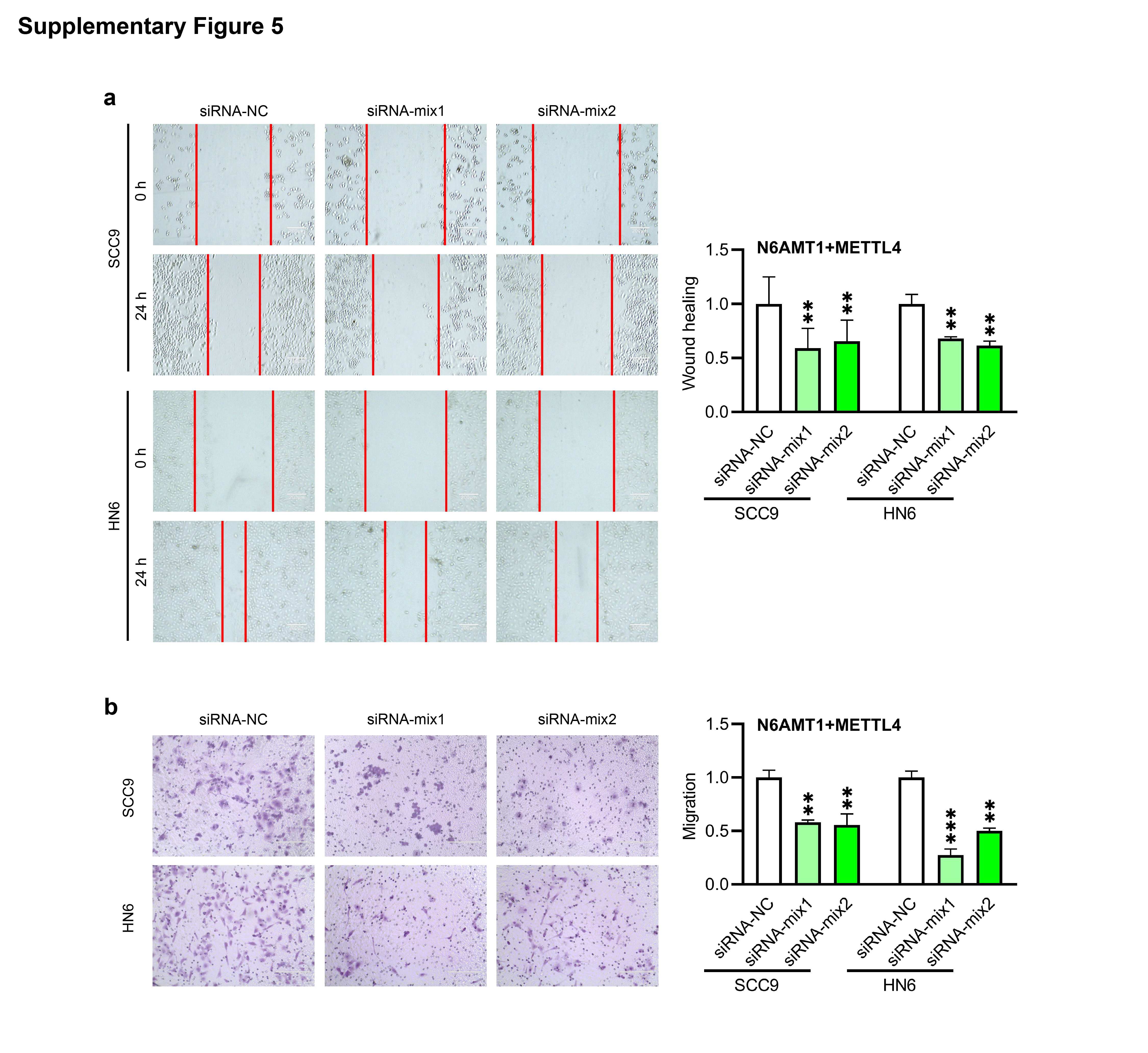


**Supplementary Figure 5.** Double knockdown of *N6AMT1* and *METTL4* led to inhibited cell migration. (a) Wound healing assay of *N6AMT1* and *METTL4* double knockdown cells. Cell images were taken and analyzed, at 0h and 24h after transfection. Scale bar, 200 μm. (b) Transwell assay of the double knockdown cells. Cell images were taken and analyzed, at 24h after transfection. Scale bar, 200 μm.


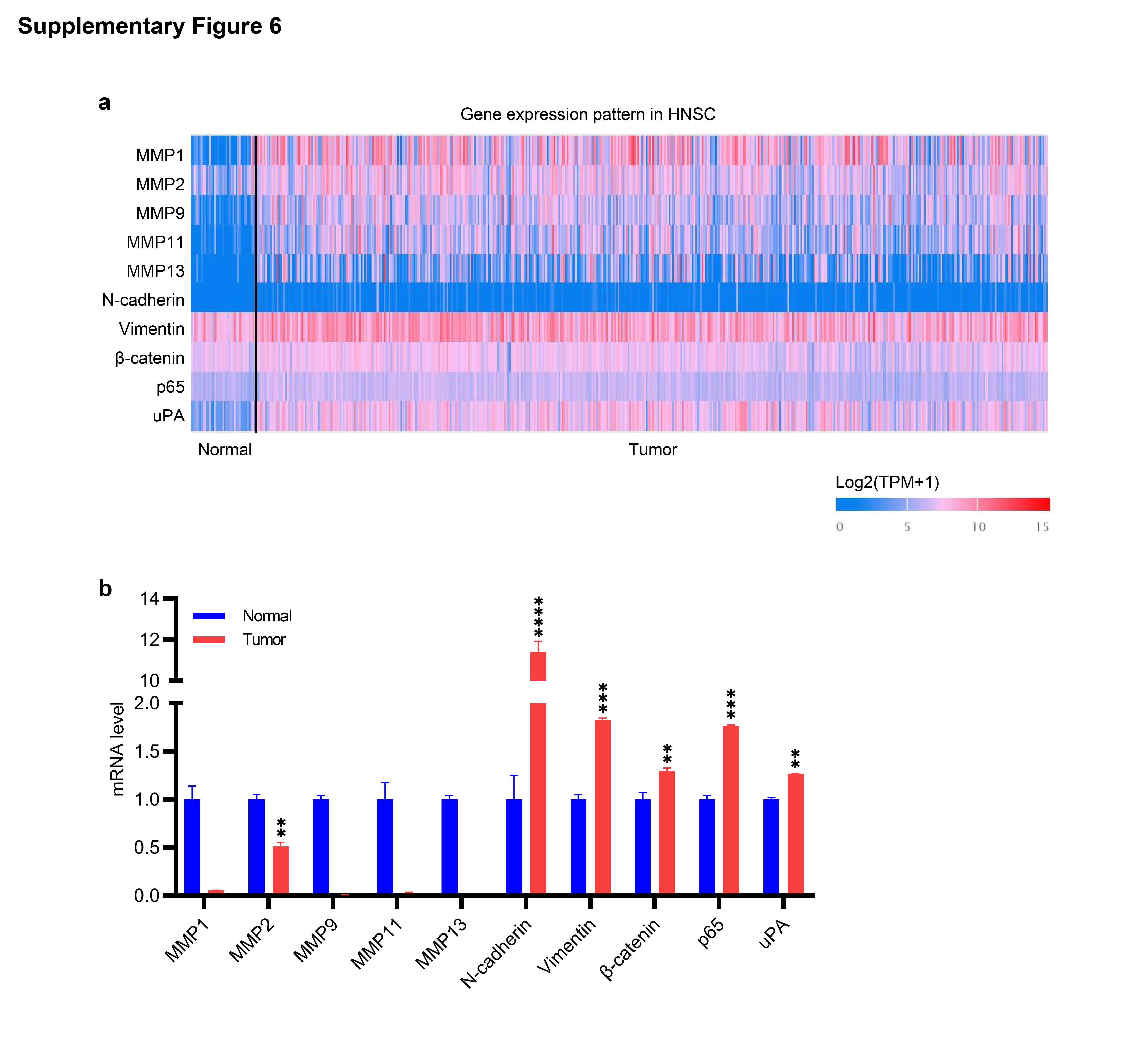


**Supplementary Figure 6.** Expression of the candidate gene set. (a) Heatmap showing gene expression levels of the HNSC and normal samples collected by TCGA database. (b) Levels of the candidate gene in tongue tumor and control tissues.


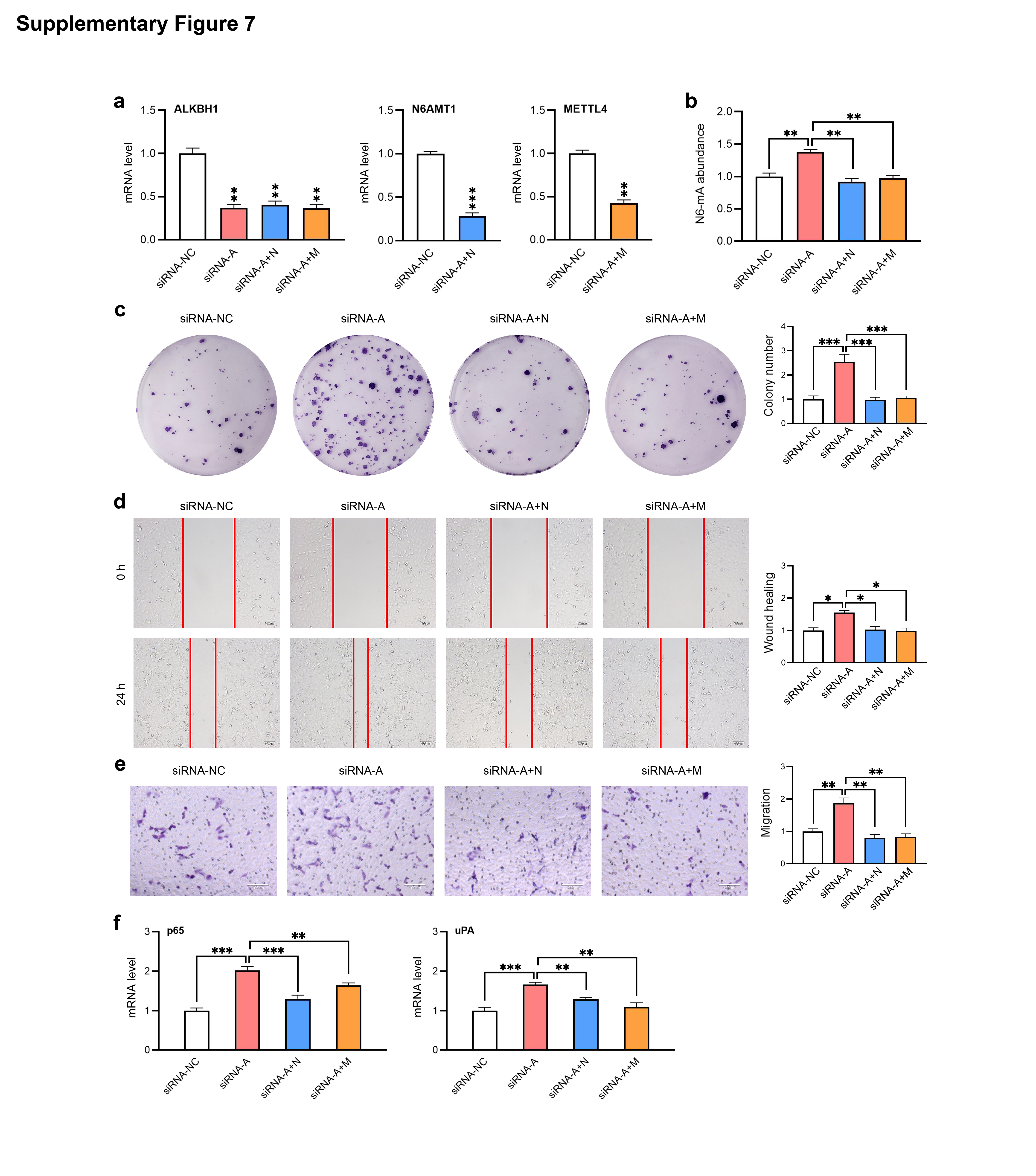


**Supplementary Figure 7.** Rescue assays performed with HN6 cells.

(a) Gene knockdown of *ALKBH1*, or double knockdown of *ALKBH1+N6AMT1, ALKBH1+METTL4*. (b) 6mA levels of single or double-knockdown cells were examined by HPLC-MS/MS. (c) Colony formation of gene knockdown cells. Both the images and quantified data are presented. (d) Wound healing of gene knockdown cells. Scale bar, 100 μm. (e) Transwell assay of gene knockdown cells. Scale bar, 100 μm. (f) Gene expression of *p65* and *uPA*

| **Supplementary Table 1. Primer sequences used in the RT-qPCR** | |
| --- | --- |
| Gene symbol | Primer sequence (5′-3′) |
| N6AMT1-F | CGGGAAGTCATGGACAGGTT |
| N6AMT1-R | GGAAAGTGCAGTGGTTCCTTG |
| METTL4-F | ATGTCTGTGGTACACCAGTTGTC |
| METTL4-R | TCAGCTTCCAGACTCCACAGC |
| ALKBH1-F | AAACTGCGTTGGGTGACCGTA |
| ALKBH1-R | CTTCAGCTCGGAAATCCTCAAATC |
| PCNA-F | GCGTGAACCTCACCAGTATGT |
| PCNA-R | TCTTCGGCCCTTAGTGTAATGAT |
| MMP1-F | AAAATTACACGCCAGATTTGCC |
| MMP1-R | GGTGTGACATTACTCCAGAGTTG |
| MMP2-F | TACAGGATCATTGGCTACACACC |
| MMP2-R | GGTCACATCGCTCCAGACT |
| MMP9-F | TGTACCGCTATGGTTACACTCG |
| MMP9-R | GGCAGGGACAGTTGCTTCT |
| MMP11-F | CCGCAACCGACAGAAGAGG |
| MMP11-R | ATCGCTCCATACCTTTAGGGC |
| MMP13-F | TCCTGATGTGGGTGAATACAATG |
| MMP13-R | GCCATCGTGAAGTCTGGTAAAAT |
| N-cadherin-F | AGCCAACCTTAACTGAGGAGT |
| N-cadherin-R | GGCAAGTTGATTGGAGGGATG |
| Vimentin-F | AGTCCACTGAGTACCGGAGAC |
| Vimentin-R | CATTTCACGCATCTGGCGTTC |
| β-catenin-F | AGCTTCCAGACACGCTATCAT |
| β-catenin-R | CGGTACAACGAGCTGTTTCTAC |
| p65-F | GTGGGGACTACGACCTGAATG |
| p65-R | GGGGCACGATTGTCAAAGATG |
| uPA-F | GGGAATGGTCACTTTTACCGAG |
| uPA-R | GGGCATGGTACGTTTGCTG |
| β-actin-F | GAGCACAGAGCCTCGCCTTT |
| β-actin-R | ATCCTTCTGACCCATGCCCA |
