# Supplementary material for: The enhanced genomic 6 mA metabolism contributes to the proliferation and migration of TSCC cells
Source: Int J Oral Sci. 2022 Feb 17;14:11. doi: 10.1038/s41368-022-00161-9 (PMC8854414; doi:10.1038/s41368-022-00161-9)
Supplement: Supplementary file 2 — read me [file 41368_2022_161_MOESM2_ESM.docx]

The supplementary information file contains supplementary figure 1-7 and supplementary table 1.
